# Early unrecognised SARS-CoV-2 introductions shaped the first pandemic wave, Sweden, 2020

**DOI:** 10.2807/1560-7917.ES.2024.29.41.2400021

**Published:** 2024-10-10

**Authors:** Robert Dyrdak, Emma B Hodcroft, Sandra Broddesson, Malin Grabbe, Hildur Franklin, Magnus Gisslén, Maricris E Holm, Magnus Lindh, Joanna Nederby-Öhd, Johan Ringlander, Martin Sundqvist, Richard A Neher, Jan Albert

**Affiliations:** 1 Department of Clinical Microbiology, Karolinska University Hospital, Stockholm, Sweden; 2 Department of Microbiology, Tumor and Cell Biology, Karolinska Institutet, Stockholm, Sweden; 3 Institute for Social and Preventive Medicine, University of Bern, Bern, Switzerland; 4 Swiss Institute of Bioinformatics, Lausanne, Switzerland; 5 Department of Laboratory Medicine, Clinical Microbiology, Örebro University Hospital, Örebro, Sweden; 6 Department of Infectious Diseases, Institute of Biomedicine, Sahlgrenska Academy, University of Gothenburg, Gothenburg, Sweden; 7 Department of Infectious Diseases, Sahlgrenska University Hospital, Gothenburg, Sweden; 8 Public Health Agency of Sweden, Solna, Sweden; 9 Department of Clinical Microbiology, Sahlgrenska University Hospital, Gothenburg, Sweden; 10 Department of Infectious Disease Prevention and Control, Stockholm Region, Stockholm, Sweden; 11 Department of Global Public Health, Karolinska Institutet, Stockholm, Sweden; 12 Faculty of Medicine and Health, Örebro University, Örebro, Sweden; 13 Biozentrum, University of Basel, Basel, Switzerland

**Keywords:** SARS-CoV-2, COVID-19 pandemic, phylogenetics, epidemiology, viral respiratory tract infection

## Abstract

**Background:**

Despite the unprecedented measures implemented globally in early 2020 to prevent the spread of SARS-CoV-2, Sweden, as many other countries, experienced a severe first wave during the COVID-19 pandemic.

**Aim:**

We investigated the introduction and spread of SARS-CoV-2 into Sweden.

**Methods:**

We analysed stored respiratory specimens (n = 1,979), sampled 7 February–2 April 2020, by PCR for SARS-CoV-2 and sequenced PCR-positive specimens. Sequences generated from newly detected cases and stored positive specimens February–June 2020 (n = 954) were combined with sequences (Sweden: n = 730; other countries: n = 129,913) retrieved from other sources for Nextstrain clade assignment and phylogenetic analyses.

**Results:**

Twelve previously unrecognised SARS-CoV-2 cases were identified: the earliest was sampled on 3 March, 1 week before recognised community transmission. We showed an early influx of clades 20A and 20B from Italy (201/328, 61% of cases exposed abroad) and clades 19A and 20C from Austria (61/328, 19%). Clade 20C dominated the first wave (20C: 908/1,684, 54%; 20B: 438/1,684, 26%; 20A: 263/1,684, 16%), and 800 of 1,684 (48%) Swedish sequences formed a country-specific 20C cluster defined by a spike mutation (G24368T). At the regional level, the proportion of clade 20C sequences correlated with an earlier weighted mean date of COVID-19 deaths.

**Conclusion:**

Community transmission in Sweden started when mitigation efforts still focused on preventing influx. This created a transmission advantage for clade 20C, likely introduced from ongoing cryptic spread in Austria. Therefore, pandemic preparedness should have a comprehensive approach, including capacity for large-scale diagnostics to allow early detection of travel-related cases and community transmission.

**Key public health message:**

**What did you want to address in this study?**

We wanted to analyse how SARS-CoV-2, the virus causing COVID-19, was introduced and thereafter spread in Sweden during the first months of the pandemic in 2020. The results may inform preparedness for future pandemics.

**What have we learnt from this study?**

Community transmission of SARS-CoV-2 in Sweden began at least 1 week earlier than previously appreciated. Due to limited capacity, testing was prioritised for travellers from northern Italy. Thus, early spread of virus variants from other regions was missed. Swedish regions with a higher share of one such variant had an earlier peak and a higher proportion of COVID-19 deaths.

**What are the implications of your findings for public health?**

During rapid global spread of a new pathogen, such as during the first wave of COVID-19 pandemic, areas with ongoing transmission are often identified in hindsight, which complicates geographically targeted prevention efforts. Therefore, pandemic preparedness should have a comprehensive approach, including capacity for large-scale diagnostics to allow early detection of travel-related cases and community transmission.

## Introduction

The betacoronavirus severe acute respiratory syndrome coronavirus 2 (SARS-CoV-2) is the cause of COVID-19 and was first detected in Wuhan, Hubei province, China, in December 2019. The virus spreads by the respiratory route, and the disease severity ranges from mild respiratory illness to life-threatening respiratory failure. Despite unprecedented preventive measures, the virus rapidly spread to the rest of the world and the outbreak was characterised as a pandemic by the World Health Organization (WHO) on 11 March 2020. Molecular surveillance of SARS-CoV-2 has allowed investigations of the dissemination into Europe and the United States (US) [1,2]. Here, we investigate how SARS-CoV-2 entered and spread in Sweden during the first pandemic wave, which in this study was defined as the period between 31 January and 1 June 2020.

During the first weeks of March 2020, the number of diagnosed SARS-CoV-2 cases and reported deaths of patients with a COVID-19 diagnosis, hereinafter called COVID-19 deaths, in Sweden increased rapidly, see Supplementary Table S1 for weekly number of cases and deaths. This may have been due to several nonexclusive reasons, including early cryptic virus circulation, a large influx of infected individuals or high-level community transmission. Understanding how these factors affected the first pandemic wave could inform future mitigation strategies. Due to limited capacity, testing was targeted at travellers returning from certain countries or regions, defined as risk areas by the Public Health Agency of Sweden (PHAS). Relevant to this study, northern Italy was labelled as a risk area from 24 February onwards and the Austrian Tyrol region from 9 March. For a more detailed description of the management of the pandemic in Sweden, see Supplementary Table S1 and Tegnell et al. [3]. For example, the Swedish government at the end of March instigated restrictions on the size of public gatherings, crowding in public spaces and visits to nursing homes.

Indications of early cryptic circulation of SARS-CoV-2 have been reported from several countries in studies based on molecular epidemiology [4-7], wastewater surveillance [8-10] and mathematical modelling [11]. Concerning the travel-associated dispersal of the virus in Europe during the first pandemic wave, several reports indicate an important contribution from early and partly cryptic virus circulation in northern Italy and Austria [4,11-15].

In this study, we have investigated the molecular epidemiology of the first pandemic wave in Sweden. The question of an early cryptic SARS-CoV-2 circulation was addressed by retrospective PCR testing for SARS-CoV-2 of stored respiratory specimens collected during the first months of 2020. The spread of SARS-CoV-2 into and within Sweden was investigated by phylogenetic analyses. Finally, we correlated the number of COVID-19 deaths across regions in Sweden with the prevalence estimates of different SARS-CoV-2 variants.

## Methods

### Retrospective PCR testing for SARS-CoV-2

Analysis for SARS-CoV-2 RNA was performed on stored remnants of 1,979 respiratory specimens that had been submitted for routine clinical molecular diagnostics for other respiratory pathogens to the Department of Clinical Microbiology at Karolinska University Hospital, Stockholm, Sweden between 7 February and 2 April 2020. The specimens had not previously been analysed for SARS-CoV-2, because the treating physician had only requested diagnostics for other respiratory pathogens, which in part may have been due to limited SARS-CoV-2 testing capacity during the first weeks of the pandemic, see Supplementary Table S1 for testing guidelines during the first pandemic wave. Most of these specimens were nasopharyngeal swabs. The laboratory catchment area includes six of the seven emergency hospitals and approximately half of the general practitioners (GPs) in the Stockholm Region that has a population of 2.4 million, i.e. almost a quarter of the Swedish population. The metadata associated with the specimens were patient identification code, age, sex, collection date and referring unit. We included all available respiratory specimens with the following exceptions: patients with a known SARS-CoV-2 detection in another specimen, sampling outside of the Stockholm Region, a refusal to use the stored specimen for research purposes and patients aged < 5 years. The age criterion was chosen because early cryptic SARS-CoV-2 was deemed unlikely to be detected in the preschool population. Randomisation was not used because we tested a high proportion of the specimens that met the inclusion criteria (1,979/2,338; 85%).

The specimens were analysed in pools of 10 using the TaqPath COVID-19 CE-IVD RT-PCR Kit (cat. A48067, Thermo Fisher Scientific, Waltham, US). A pool was considered reactive if at least one of the three SARS-CoV-2 gene targets (ORF1ab, N, S) was detected. Reactive pools were split, and specimens were analysed individually. A specimen was considered positive if at least two targets were detected and indeterminate if one target was detected. Illumina sequencing was attempted for all positive specimens. See Supplement Methods for details on protocols and specimens.

### SARS-CoV-2 whole genome sequences

**Figure 1:**
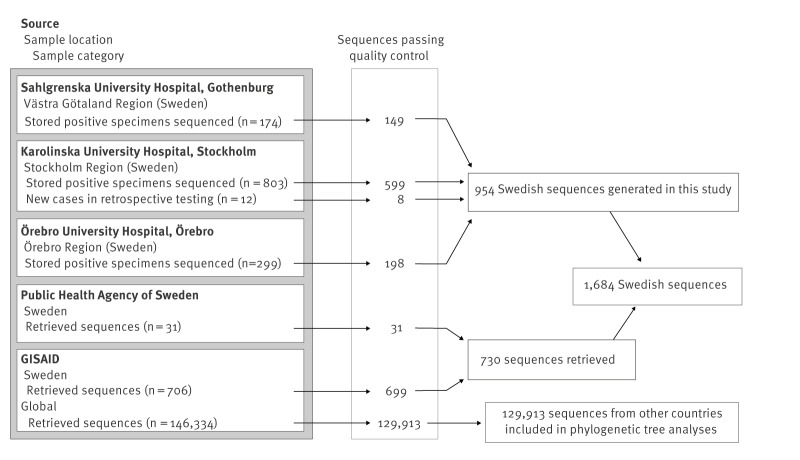
Sources of severe acute respiratory syndrome coronavirus 2 (SARS-CoV-2) sequences and number of sequences included, Sweden (n = 1,684) and other countries (n = 129,913), December 2019–June 2020

The final dataset consisted of 1,684 Swedish SARS-CoV-2 sequences, of which 954 were generated in this study, and 129,913 sequences were from other countries (Figure 1). As described in detail in Supplement Methods, we attempted whole genome sequencing on 1,288 stored SARS-CoV-2-positive respiratory specimens collected between 26 February and 1 June 2020, in the participating clinical microbiology laboratories in Gothenburg (Västra Götaland Region), Stockholm and Örebro (Figure 1). During this period, the laboratories used several different commercial and laboratory-developed methods for SARS-CoV-2 PCR testing. Guidelines for SARS-CoV-2 PCR testing were issued by the PHAS and updated several times based on the epidemiological situation and testing capacity. Randomisation was not used to select specimens for sequencing, instead specimens were chosen to represent patients with and without travel history, as well as patients from hospitals, outpatient clinics, nursing homes and primary care settings. The 1,288 specimens were subjected to RNA extraction, SARS-CoV-2 amplification, next generation sequencing and bioinformatic processing into consensus SARS-CoV-2 whole genome sequences at the participating laboratories using local protocols. All consensus sequences were analysed by Nextclade version 2.4.0 [16] using Wuhan-Hu-1 (MN908947) as a reference, yielding quality control scores, clade assignment (Nextstrain clades 19A, 19B, 20A, etc.) and mutations relative to the reference sequence. Sequences with a Nextclade quality score classified as good and within four interquartile ranges in root-to-tip vs time regression were retained for further analysis (n = 954) (Figure 1). From the GISAID repository [17], we downloaded all sequences with collection date up to 1 June 2020 (as available on 22 August 2022, https://gisaid.org/EPI_SET_231220zv, Supplementary Table S2). This dataset comprised 706 Swedish sequences and 146,334 sequences from other countries (Figure 1). Thirty-one additional Swedish sequences were obtained from the PHAS. Sequences with a Nextclade quality score classified as good were retained, and Swedish sequences underwent the same quality selection of root-to-tip vs time regression as described above, whereas sequences from other countries assigned to clades other than 19A–B and 20A–D were considered to have misannotated collection dates or other typing issues and were excluded, as other clades appeared after June 2020.

### Phylogenetic analysis

Two phylogenetic trees were constructed: one divergence tree that contained all 1,684 Swedish sequences and all 129,913 sequences from other countries passing the quality controls and one down-sampled time-scaled tree containing the 1,684 Swedish sequences and 1,199 sequences from other countries obtained by random subsampling of the dataset with and without priority for proximity to Swedish sequences. The trees were constructed using the SARS-CoV-2 workflow available in the Nextstrain Augur pipeline [18]: sequence alignment was performed using Nextalign version 1.4.0 with Wuhan-Hu-1 as the reference; parsimony maximum-likelihood trees were constructed using MAPLE version 0.2.1 [19]; and for the down-sampled dataset we used TreeTime version 0.8.4 [20] to construct a time-scaled phylogeny and to perform root-to-tip vs time regression. The topology of the Swedish sequences was inspected in the time-scaled tree and Swedish clusters were investigated by extracting subphylogenies (subtrees) from the divergence tree using matUtils [21]. Tree visualisation was done using the Nextstrain Auspice tool.

### Metadata and national statistics

The metadata for the Swedish sequences included the reported country or location of the infection and the specimen collection date. Information of the likely country or location where patients were infected was obtained by an excerpt from SmiNet. SmiNet is the Swedish national electronic notification system where treating physicians and clinical laboratories are to report notifiable diseases to the regional Infectious Disease Control Authorities and the PHAS. Additional contact tracing records were obtained from the regional Infectious Disease Control Authorities in the Stockholm, Västra Götaland and Örebro Regions. For the 12 previously unrecognised cases detected by the retrospective testing, we asked the treating physicians or the referring units to review the medical record for any travel history. The metadata for the sequences in GISAID were those available upon download, i.e. collection date and country of sampling.

The number of recorded COVID-19 deaths until 7 June 2020 were obtained from public data from the PHAS [22].

### Statistical analysis

The mean week of recorded COVID-19 deaths in a region was calculated as a weighted mean date, where the date (calendar week) was weighted by the number of reported deaths (i.e. the sum of the products of week number and number of reported deaths that week, divided by the total number of recorded deaths). In the analysis of molecular epidemiology in relation to COVID-19 deaths, all cases sampled later than 29 March were assumed to have been infected in Sweden. A sensitivity analysis was also done using all exposure data as reported.

Linear regression modelling and the Wilcoxon rank sum test were performed using Stata/IC 15.1 (StataCorp LLC, College Station, US). A p value < 0.05 was considered statistically significant.

## Results

### Early SARS-CoV-2 cases detected in retrospective testing

**Figure 2:**
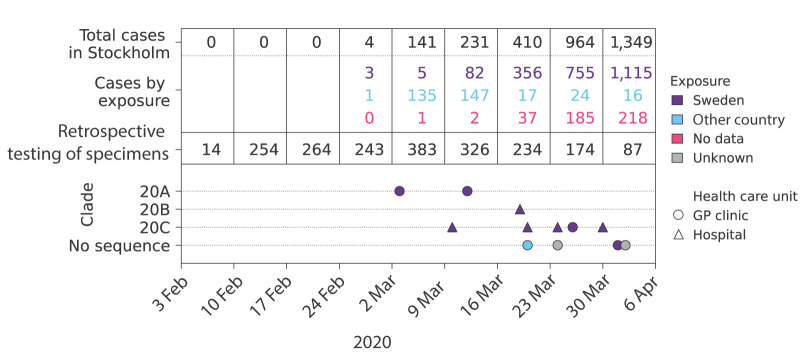
Retrospective PCR testing of stored respiratory specimens for severe acute respiratory syndrome coronavirus 2 (SARS-CoV-2), by date and exposure, Stockholm Region, Sweden, February–April 2020 (n = 1,979)

We identified 18 positive and three indeterminate specimens among the 1,979 stored respiratory specimens. The 18 positive specimens corresponded to 17 patients, of whom 12 were previously unrecognised SARS-CoV-2 cases. Seven of these new cases had been sampled at GP clinics and five at hospitals (Figure 2).

GP: general practitioner.

Newly detected cases are indicated by circles and triangles. Colours of numbers and symbols for new cases indicate exposure. No data indicates a lack of data, whereas Unknown indicates an unresolved place of exposure. Clade refers to Nextstrain clade assignment. No sequence indicates that sequencing was unsuccessful. The X axis shows the sampling date. The number of previously reported cases in Stockholm and the number of specimens tested for this study are aggregated by calendar week.

Specimens tested and the clade markers are from the retrospective testing and total cases and cases by exposure are from Public Health Agency of Sweden and the regional Infectious Disease Control Authority in Stockholm.

Nine of the new cases had no travel history and no known contact with other SARS-CoV-2 cases. One of these cases was sampled on 3 March. Previously, the first known cases in the Stockholm Region were sampled on 8 March. Thus, our novel case sampled on 3 March predated the previously recognised onset of community transmission by almost a week. Whole genome sequencing of SARS-CoV-2 was successful for specimens from eight of the new cases (Figure 2). A clade 20A virus was characterised in the earliest case, whereas clade 20C was the most common clade (n = 5).

### Characteristics of Swedish SARS-CoV-2 sequences

**Figure 3:**
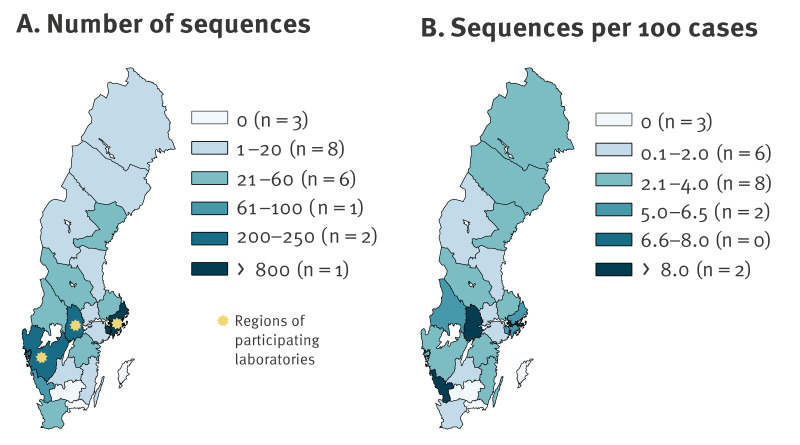
Number of sequences of severe acute respiratory syndrome coronavirus 2 (SARS-CoV-2), by region, Sweden, January–June 2020 (n = 1,684)

Of the 1,684 sequences analysed, 954 were generated in this study, 699 were retrieved from GISAID and 31 were obtained from the PHAS (Figure 1). The dataset included sequences from 18 of the 21 Swedish regions, and approximately half (n = 823, 49%) were collected in the Stockholm Region. Nationally, there were 4.3 sequences per 100 reported cases of SARS-CoV-2 infection during the first wave. The highest proportions of sequenced specimens from cases were from the regions of Örebro, Halland and Stockholm, with 11.8, 8.6 and 6.2 sequences per 100 confirmed cases, respectively (Figure 3), see Supplementary Table S3 for statistics for all regions. According to the Nextstrain classification system, most of the 1,684 Swedish sequences belonged to clade 20C (n = 908, 54%), followed by 20B (n = 438, 26%), 20A (n = 263, 16%), 19A (n = 66, 4%), 20D (n = 5, 0.3%) and 19B (n = 4, 0.2%) (Table).

Panel A. Absolute number of SARS-CoV-2 sequences, until 1 June 2020.

Panel B. SARS-CoV-2 sequences per 100 reported cases of SARS-CoV-2 infection, until 1 June 2020.

The regions of the three participating clinical laboratories in Gothenburg, Stockholm and Örebro are marked with yellow stars.

**Table:** Number of sequences of severe acute respiratory syndrome coronavirus 2 (SARS-CoV-2), by case exposure and virus clade (Nextstrain clade assignment), Sweden, January–June 2020 (n = 1,684)

Exposure		Clade						Total
Category	Country	19A	19B	20A	20B	20C	20D	
Domestic	Sweden	22	3	141	189	722	0	1,077
Abroad	Austria	22	0	8	2	39	0	71
Abroad	Italy	5	0	79	122	1	5	212
Abroad	Other countries	13	0	10	9	13	0	45
No data	No data	4	1	25	116	133	0	279
Total		66	4	263	438	908	5	1,684

### SARS-CoV-2 clades differed between cases exposed abroad and in Sweden

**Figure 4:**
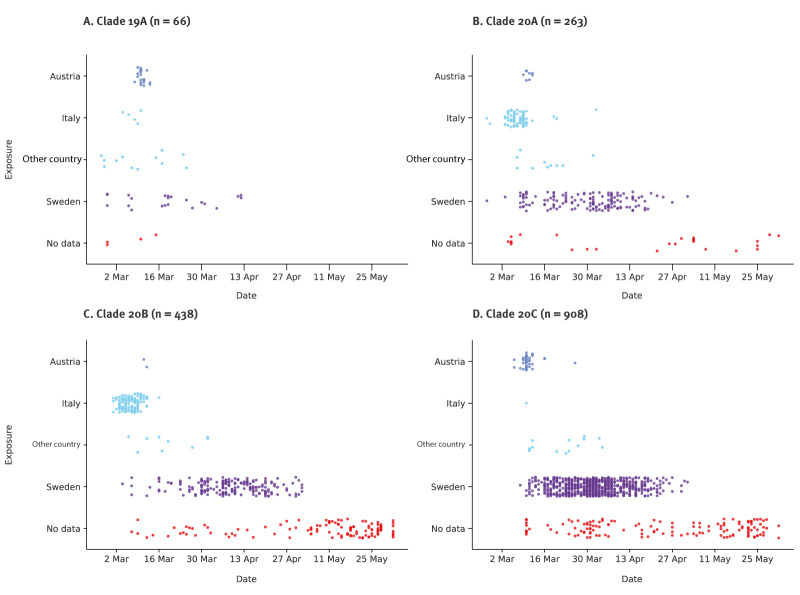
Sequences of severe acute respiratory syndrome coronavirus 2 (SARS-CoV-2), by genetic clade and patient exposure, Sweden, February–June 2020 (n = 1,675)^a^

^a^ The earliest case (31 January, Clade 19A, Other country) is truncated from the figure.

Information about the likely country of infection was available for 1,405 of 1,684 (83%) sequenced Swedish cases (Table). Among cases with such information, 328 (23%) were reported to have been exposed abroad. The most common countries of exposure were Italy (n = 212, 65%) and Austria (n = 71, 22%) (Figure 4, Table). Twenty-nine (9%) cases were exposed in other European countries, six (2%) in the Middle East and 10 (3%) in other countries outside Europe. Among cases exposed abroad, Italy was the most common country of exposure for cases with clades 20B (122/133 cases, 92%) and 20A (79/97 cases, 81%), whereas Austria was the most common country for cases infected with clades 20C (39/53 cases) and 19A (22/40 cases) (Table). Most cases without information about the country of infection were sampled late during the first wave, thus, the infections were likely acquired in Sweden.

The panels show the sampling dates of cases for the four main sequence clades in Sweden 26 February–1 June 2020. The cases are coloured by country of exposure (Sweden, Italy, Austria and other countries).

There were marked differences in the proportion of SARS-CoV-2 clades between infections acquired abroad vs domestically. Thus, 230 of 701 cases (33%) infected with clades 20A and cases in Sweden were exposed in China and Iran and were infected with clade 19A, there were also early cases infected with clades 20A and 20B who were exposed in Italy (Figure 4).

### A few early virus introductions accounted for most domestic transmission

We used phylogenetic tree analyses to identify routes and sources of SARS-CoV-2 influx and onward transmission in Sweden. Clusters consisting entirely or mostly of Swedish sequences were identified using the 129,913 international GISAID sequences as background data. These international sequences originated from 149 countries, of which 61,362 (47%) were from Europe. Two countries accounted for more than half of the sequences (US and the United Kingdom (UK), 36,933 (28% of the sequences) and 33,752 (26%), respectively).

**Figure 5:**
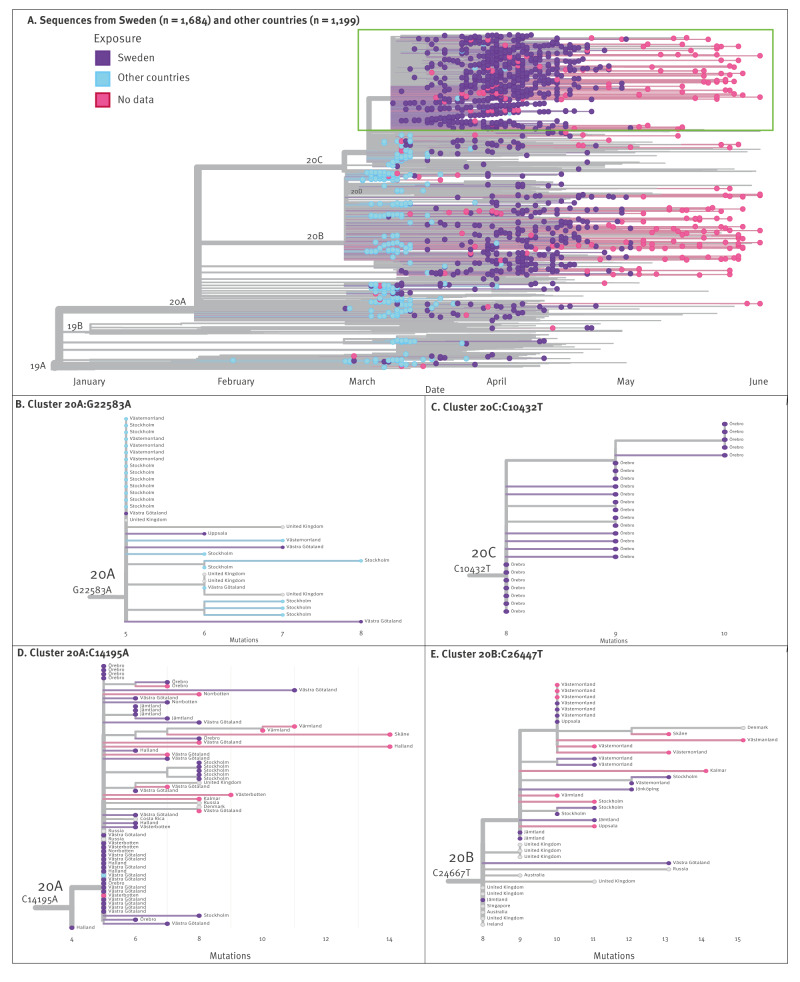
Phylogenetic tree of sequences and selected clusters of severe acute respiratory syndrome coronavirus 2 (SARS-CoV-2), Sweden (n = 1,684) and other countries (n = 1,199), January–June 2020

In the time-scaled phylogenetic tree, 140/328 (43%) of the sequences from the Swedish cases exposed abroad had no identical or descendent sequences in Sweden, suggesting limited or no onward domestic transmission (Figure 5A). Instead, a few virus introductions accounted for most domestic transmissions and gave rise to one big and several smaller Swedish clusters. Thus, almost half (800/1,684, 48%) of all Swedish sequences formed a cluster within clade 20C defined by the mutation G24368T (20C:G24368T) which encodes the spike substitution D936Y (Figure 5A). Of these, 252 sequences (32%) belonged to the root of this variant. The 20C:G24368T cluster dominated among domestically exposed cases (679/1,077, 63%). Globally, the earliest detection of a 20C:G24368T variant was on 8 March in Sweden, followed by Denmark and the UK on the next day and within a week in the US, Norway, Saudi Arabia and the Danish territory Faroe Islands. However, the variant was more dominant in Sweden than in any of these countries (Norway: 59/391, 15%; Denmark 33/1,737, 2%; UK: 400/33,752, 1%, Saudi Arabia: 2/604, 0.3% and US: 41/36,933, 0.1%). The 20C:G24368T variant also became dominant in Finland (400/744, 54%) where it was detected on 23 March, i.e. > 2 weeks later than in Sweden. Albeit Austria was the most common country of exposure among the travel-related Swedish cases with clade 20C, the 20C:G24368T variant was not detected among these cases.

Tips are coloured by place of exposure (Sweden, abroad, and no data).

Panel A. Time-scaled phylogeny of Swedish sequences (coloured dots) and international sequences (no dots). The X axis shows the sampling date. The green box indicates the cluster 20C:G24368T which constituted 800 (48%) of Swedish sequences.

Panel B. Cluster 20A:G22583A, detected in several Swedish regions but with limited detected onwards transmission.

Panel C. A subcluster within the large 20C:G24368T cluster detected only in the Örebro Region.

Panel D. A Swedish cluster dispersed into several regions with local subclusters.

Panel E. A Swedish cluster dispersed into several regions with local subclusters.

The X axis in Panels B–E shows the divergence (number of nucleotide substitutions) of strains from reference sequence Wuhan-Hu-1. The tip labels denote the region of sampling for Swedish sequences and the country of sampling for sequences from other countries.

Figure 5B shows cluster 20A:G25583A, in which all the travel-related Swedish cases were exposed in Italy, yet in the international dataset there was no Italian 20A sequence with the cluster-defining mutation G25583A. Like for cluster 20C:G24368T, this may illustrate a lack of sequences from source regions despite the large size of the international reference dataset (n = 129,913). For the 1,077 cases with domestic exposure, there was information on exposure at region level for 678 (63%) of the cases, and of these, 653 (96%) were reported to have been exposed within their region of diagnosis. In Figure 5, the panels C-E illustrate that there were some domestic clusters with cases from a single region, as well as clusters dispersed over several regions.

### The proportion of clade 20C as proxy for early community transmission

**Figure 6:**
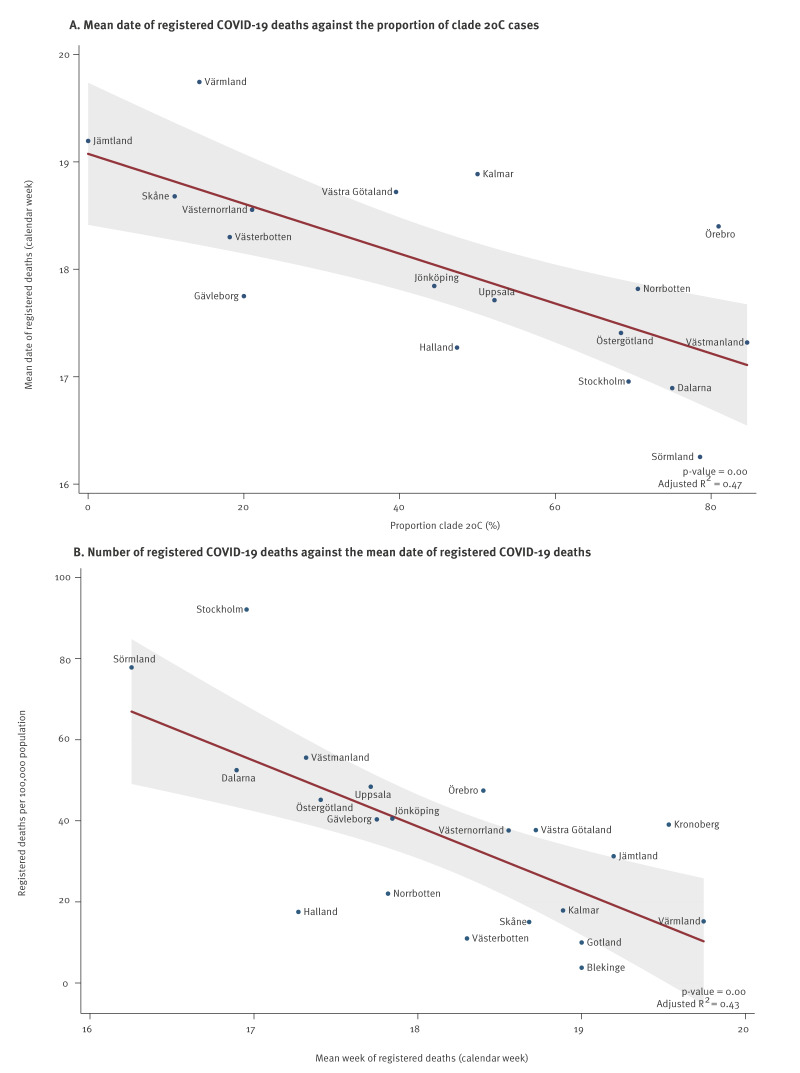
Registered deaths of patients with a COVID-19 diagnosis (n = 4,867), by the proportion of clade 20C severe acute respiratory syndrome coronavirus 2 (SARS-CoV-2) among domestic cases (A) and cumulative number of registered deaths of patients with a COVID-19 diagnosis, by mean date of registered deaths of patients with a COVID-19 diagnosis (B), Sweden, January–June 2020

Overall, clade 20C represented 722 of 1,077 (67%) of Swedish cases contracted in Sweden, but the proportion of clade 20C infections differed substantially between regions (range: 0–85%). A higher proportion of domestically acquired clade 20C infections correlated with an earlier weighted mean date of registered deaths (p = 0.001, Figure 6A). An earlier mean date of registered deaths in turn was correlated with a higher total number of registered COVID-19 deaths during the first pandemic wave (p = 0.001, Figure 6B). However, in a multiple regression analysis with the cumulative number of registered deaths as the outcome variable, neither the proportion of clade 20C (p = 0.36) nor the mean date of registered deaths (p = 0.065) remained a statistically significant coefficient. This suggests that there was a multicollinearity between these two explanatory variables, such that the proportion of clade 20C infections acted a proxy for an early start of community transmission as well as a higher number of registered COVID-19 deaths. In a sensitivity analysis that used the registered exposure status (instead of an assumption of exposure in Sweden for all cases diagnosed after 29 March), the proportion of clade 20C infections remained significantly correlated with the mean date of registered deaths (p = 0.032), plotted in Supplementary Figure S1.

R^2^: coefficient of determination.

A higher proportion of clade 20C correlated with an earlier mean date of COVID-19 deaths. An earlier mean date of registered COVID-19 deaths correlated with a higher cumulative number of registered deaths. All cases sampled later than 29 March 2020 were considered to have been infected in Sweden. The mean week of reported COVID-19 deaths in a region was calculated as a weighted mean date, where the date (calendar week) was weighted by the number of reported deaths.

Line:  fitted mean of linear regression; shaded area:  95% confidence interval of the predicted mean.

## Discussion

We have investigated the molecular epidemiology of the first SARS-CoV-2 pandemic wave in Sweden. Italy and Austria were the most common countries of exposure among cases reported to have been infected abroad. Variants of SARS-CoV-2 belonging to Nextstrain clades 20B and 20A predominated among travellers from Italy and clades 20C and 19A among travellers from Austria. Although exposure in Italy was three times more common among the cases with sequenced specimens than exposure in Austria, clade 20C dominated among Swedish sequences (n = 908, 54%). The proportion of clade 20C differed between regions of Sweden and correlated with the reported per capita number of COVID-19 deaths, indicating that the proportion of clade 20C acted a proxy for an early cryptic introduction and dissemination of SARS-CoV-2 in Sweden.

The detection of early domestic infections through retrospective testing indicated that community transmission in Sweden started at least a week earlier than previously estimated. Similar findings have been reported from other countries [4,5,7,12,23]. In line with this, the Omicron (Phylogenetic Assignment of Named Global Outbreak (Pango) lineage designation B.1.1.529) variant had already spread to secondary locations when it was detected in Botswana and South Africa in November 2021 [24]. Thus, attempts to prevent influx of new SARS-CoV-2 variants have often been initiated too late, rendering them largely ineffective.

In the phylogenetic tree, 140 of 328 (43%) of sequences from Swedish cases with exposure abroad did not have descendants in Sweden. This agrees with other reports on the epidemiology of the first pandemic wave, where a small number of introductions were responsible for most downstream transmissions and early seeding resulted in large clusters [7,12,23,25-27]. However, in Sweden, clade 20C became dominant despite the earlier introductions of clades 19A, 20A, and 20B. Indeed, among returning travellers sequenced in this study, there were more cases infected with clades 20A (n = 97) and 20B (n = 133), respectively, than with 20C (n = 53). This might be explained by the earlier identification of Italy as a risk area compared with Austria. The Austrian Alps, and in particular the Ischgl ski resort in the Tyrol region, acted as a hub for transmission and dispersal of clade 20C [28] and were the source of introductions into Iceland, Denmark, Norway, Germany and Switzerland [13-15,28-30]. Likewise, Austria was the most common travel destination among Swedish cases infected with clade 20C and exposure abroad. Our results suggest that targeted testing, which was prioritised for travellers from northern Italy, and public awareness about ongoing spread of SARS-CoV-2 in northern Italy may have reduced onwards transmission of clades 20A and 20B in Sweden. In contrast, the introduction and onwards spread of clade 20C was less affected. Our study illustrates that risk areas may have been identified too late for effective preventive measures. Early establishment of large-scale diagnostic capacity and wastewater-based surveillance could enable timely and unbiased detection of travel-related cases and community transmission.

The largest cluster in clade 20C was defined by the G24368T mutation. The root of this 20C:G24368T variant was by far the most common haplotype in Sweden which could indicate that a superspreading event or events may have contributed to the spread of this variant. Although 20C:G24368T was not detected among Swedish cases exposed in Austria, the almost simultaneous appearance of the variant in Sweden, the UK and Denmark in the second week of March suggests a parallel influx from a common source, e.g. the Austrian Alps. The variant 20C:G24368T has not been reported among Austrian sequences which might be due to limited sampling of early Austrian cases or that transmission of the variant primarily occurred among visiting tourists. The dominance of the variant 20C:G24368T in Sweden was likely due to a founder effect, rather than an increased transmissibility of the variant as no similar dominance occurred in the UK or Denmark. Although there was no formal border closure in Sweden, the marked decline in international travel would have accentuated such a founder effect. Moreover, the spike substitution D936Y (encoded by the G24368T mutation) does not result in increased infectivity in vitro [31]. It has been reported that the G24368T mutation was positively selected in Sweden [32], but we believe that this was due to an early and undetected entry that gave the 20C:G24368T variant a transmission advantage.

We observed a correlation between a high proportion of clade 20C sequences and an earlier peak of registered COVID-19 deaths. It is likely that individuals infected with clade 20C more often may have been unaware of their infection status and therefore contributed to onward transmission. Thus, the proportion of clade 20C appears to be a proxy for an early cryptic introduction and dissemination of SARS-CoV-2 in different regions in Sweden. It happened to be clade 20C but could have been any other clade. An earlier peak of COVID-19 deaths (at the regional level) also correlated with a higher per capita cumulative number of such deaths, which may have several non-exclusive explanations factors. If the number of registered deaths reflects overall community transmission, it seems that regions with lower proportion of the 20C clade also had fewer infections per capita. It is also likely that hospital care improved over time. Our study does not answer why the proportion of clade 20C varied substantially across Swedish regions.

A limitation of our study is that only a fraction of all Swedish SARS-CoV-2 cases during the first pandemic wave were diagnosed, and even fewer were sequenced. Also, testing recommendations that targeted travellers from specific regions, such as northern Italy, created a selection bias among cases exposed abroad. Seroprevalence studies by the PHAS showed that 5% of individuals sampled in the week of 1 June 2020 were seropositive which indicates that approximately half a million SARS-CoV-2 infections occurred in Sweden during the first pandemic wave [33]. Consequently, the sequences in this study represent < 0.5% of the actual number of cases. However, this fraction was likely sufficient to detect major transmission patterns, in the study of SARS-CoV-2 introduction into the UK there were sequences for 0.7% of the estimated actual cases [12]. The genetic diversity of SARS-CoV-2 was limited during the first pandemic wave, making it difficult to phylogenetically estimate the number of virus introductions into Sweden. However, the contact tracing data in Stockholm showed this to be in the range of at least hundreds.

## Conclusion

During the first pandemic wave in Sweden, community transmission of SARS-CoV-2 started earlier and through different travel routes than previously assumed but was not preceded by a prolonged cryptic circulation. Clade 20C dominated among Swedish sequences but not among cases with exposure abroad which were dominated by clades 20A and 20B. A high proportion of clade 20C in a region correlated with an earlier peak of COVID-19 deaths which in turn was correlated with more per capita COVID-19 deaths. Although various factors might have contributed to this, it illustrates a likely benefit of delaying the incidence peak. The molecular epidemiology of the first pandemic wave in Sweden illustrates that risk areas may be identified too late for effective preventive measures targeted at travellers from such areas. For future pandemic preparedness, this argues for a rapid establishment of large-scale diagnostic capacity to enable early detection travel-related cases and of community transmission.

## Supplementary Data

301000 Supplementary Material
