# Supplementary material for: Early unrecognised SARS-CoV-2 introductions shaped the first pandemic wave, Sweden, 2020
Source: Euro Surveill. 2024 Oct 10;29(41):2400021. doi: 10.2807/1560-7917.ES.2024.29.41.2400021 (PMC11484920; doi:10.2807/1560-7917.ES.2024.29.41.2400021)
Supplement: Supplementary Material [file 24-00021_DYRDAK_Supplement.pdf]

Supplementary materials

This supplementary material is hosted by Eurosurveillance as supporting information alongside the article *Early unrecognized SARS-CoV-2 introductions shaped the first pandemic wave in Sweden*, on behalf of the authors, who remain responsible for the accuracy and appropriateness of the content. The same standards for ethics, copyright, attributions and permissions as for the article apply. Supplements are not edited by Eurosurveillance and the journal is not responsible for the maintenance of any links or email addresses provided therein.

Table of Contents

1. **Methods** 2
   1.1. **Retrospective SARS-CoV-2 PCR testing of stored respiratory samples** ... 2
   1.2. **Routine SARS-CoV-2 diagnostics and selection of samples for sequencing** 2
   1.3. **Phylogenetics trees** 5
2. **Figures and tables** 5
   2.1. **Table S1** 6
   2.2. **Table S2** 8
   2.3. **Table S3.** 9
   1.1. **Figure S1.** 10

References 11

1. Methods

1.1. Retrospective SARS-CoV-2 PCR testing of stored respiratory samples

Remnants of respiratory specimens submitted for clinical diagnostics had been stored at -70°C. Samples were pooled, with a pool consisting of up to 10 samples in equal proportions. Then, 25 µL of a pool was diluted 1:10 in 250 µL MagNA Pure 96 External Lysis Buffer (ELB) (cat. number 06374913001, Roche Diagnostics AB). For automated extraction and PCR setup, Hamilton STARlet KingFisher Presto was used with the MagMAX Viral/Pathogen Nucleic Acid Isolation Kit (cat. number A42352; Thermo Fisher Scientific) and the TaqPath COVID 19 CE IVD RT PCR Kit. QuantStudio 5 (Thermo Fisher Scientific/Applied Biosystems) was used for real-time PCR.

The assay contains three SARS-CoV-2 specific gene targets (ORF1ab, N, S). A pool was considered reactive if at least one of the targets had a cycle threshold (Ct) value of less than 40. Reactive pools were split and the individual samples were tested as described above in a 1:10 dilution (50 µL sample + 450 µL ELB). A sample was considered positive if at least two targets were detected and indeterminate if one target was detected. Positive samples were sequenced, performed as described below.

Randomisation was not used for sample selection because a large proportion of the sample was tested 1,979/2,338 (85%) of specimens meeting the inclusion criteria). As we did not detect any new cases in the specimens prior to 3 March, we did not progress to test specimens collected earlier than 7 February.

The specimen types were nasopharyngeal secretions (71%), sputum samples (9%), pharyngeal secretions (9%), nasopharyngeal aspirates (6%), bronchoalveolar lavages (4%), tracheal secretions (1%), and other (1%).

1.2. Routine SARS-CoV-2 diagnostics and selection of samples for sequencing

Stored respiratory specimens positive for SARS-CoV-2-RNA in clinical routine diagnostics were sequenced.

1.2.1. SARS-CoV-2 PCR diagnostics

In Gothenburg, a laboratory developed test (LDT) PCR assay was used for SARS-CoV-2 diagnostics during the study period. Nucleic extraction was performed with the MagNA Pure 96 (Roche Diagnostics) and reverse transcriptase quantitative PCR using the ABI 7300 (Thermo Fisher Scientific), targeting SARS-CoV-2 orf1ab using the primers and probe by Ringlander *et al* [1].

In Stockholm, four methods for SARS-CoV-2 PCR diagnostics were used. These were an LDT, which (i) was run with separate extraction on MagNA Pure 96 (Roche Diagnostics) and PCR on either Quantstudio 6 Flex (Thermo Fisher Scientific), or ABI 7500 (Thermo Fisher Scientific), or (ii) with integrated extraction and PCR on the NeuMoDx 288 Molecular System (Qiagen), and two commercial kits: (iii) the Cobas 6800 system (P/N: 09175431190; Roche Diagnostics), and (iv) GeneXpert (Xpert Xpress SARS-CoV-2 302-3562; Cepheid). The LDT used primers and probe targeting the RdRp and E genes by Corman *et al* [2] until 31 March 2020, and from 1 April 2020 a modified version of these from the Gothenburg laboratory was used.

In Örebro, the samples were analysed using an LDT PCR targeting the E-gene as the first-line screening tool, followed by confirmatory testing with the RdRp gene assay, using the primers and probe by Corman *et al* [2].

1.2.2. Selection of samples for sequencing

In Gothenburg, we aimed to sequence 200 samples. After identifying cases, 174 specimens eligible for sequencing were found in the biobank. Randomisation was not used for the selection but samples

were chosen to represent both hospitalized and out-patients, with and without travel history, as well as patients in nursing homes and primary care, that had not travelled.

In Stockholm, we aimed to choose 100 specimens for each week. Within these, we prioritized samples from patients with travel-history and also aimed for representation of various referring units, including nursing homes, and samples with a cycle threshold value below 30 in the in diagnostic SARS-CoV-2 PCR. Randomisation was not applied. Eventually, sequencing was attempted on 803 stored specimens.

In Örebro, we identified the first 300 SARS-CoV-2 positive patients in the Örebro Region. Sequencing was attempted on specimens with a cycle threshold value below 35 in the in the diagnostic SARS-CoV-2 PCR. Some of these specimens (n=13) had already been sequenced but the majority were sequenced in this study. Thus, specimens were selected sequencing based on sampling date.

1.2.3. Illumina

All specimens in Stockholm, and 154 of 174 specimens in Gothenburg, were sequenced using Illumina technology (Illumina). In Stockholm, samples were extracted, sequenced and bioinformatically processed at the Department of Clinical Microbiology at Karolinska University Hospital, Stockholm, Sweden, and SciLifeLab, Stockholm, Sweden; and in Gothenburg at the Department of Clinical Microbiology, and the Department of Clinical Genetics, Sahlgrenska University Hospital, Gothenburg, Sweden.

The samples were diluted 1:2 in MagNA Pure 96 External Lysis Buffer (cat. number 06374913001, Roche Diagnostics) for virus inactivation and then RNA was extracted on MagNA Pure 96 using the MagNA Pure 96 DNA and Viral NA SV Kit (cat. number 06543588001, Roche Diagnostics). cDNA was prepared using the Illumina COVIDSeq Test Kit (Document #1000000126053 v02) according to the manufacturer's instructions: 8.5 µL of RNA eluate was mixed with 8.5 µL of Elution Prime Fragment 3HC Mix HT followed by annealing at 63°C for 3 minutes and 4°C, indefinitely. Then, 0.8 µL of Reverse Transcriptase HT and 7.2 µL First Strand Mix HT were added to the annealed RNA. The protocol for cDNA synthesis was 25°C for 5 minutes, 50°C for 10 minutes, 80°C for 5 minutes, and 4°C, indefinitely.

The obtained cDNA was used for the amplification of the whole SARS-CoV-2 genome with overlapping amplicons according to ARTIC protocol version 3 (https://artic.network/ncov-2019). Two reactions per sample were set up with the following contents: 12.5 µL Illumina PCR Mix HT, 3.9 µL nuclease-free water, 3.6 µL each of COVIDSeq primer pool 1 HT and COVIDSeq primer pool 2 HT, and 5 µL cDNA. The amplification protocol was 98°C for 3 minutes and 35 cycles of 98°C for 15 seconds and 63°C for 5 minutes, then 4°C, indefinitely. After amplification, the reactions were pooled to a final volume of 30 µL.

PCR-amplified DNA was used to produce sequencing libraries with tagmentation methodology (Illumina). To each reaction mix, 10 µL Tagmentation Buffer 1 HT, 3.3 µL Enrichment BLT HT and 16.7 µL nuclease-free water were added. Samples were incubated at 55°C for 5 minutes followed by 10°C, indefinitely. After tagmentation, the amplicons were cleaned by adding 10 µL Stop Tagment Buffer 2 HT followed by incubation at room temperature for 5 minutes and magnetic separation. The magnetic beads were washed twice with Tagmentation Wash Buffer HT. Amplification of tagmented amplicons was carried out by adding 20 µL Enhanced PCR mix HT, 10 µL Index Adapters, and 20 µL nuclease-free water to each sample and incubation at 72°C for 3 minutes, 98°C for 3 minutes, 7 cycles of: 98°C for 20 seconds, 60°C for 30 seconds, 72°C for 1 minute; followed by 72°C for 3 minutes and 4°C, indefinitely. Amplicon libraries were pooled and 0.9X volume Illumina Tune Beads were added. After 5 minutes of incubation at room temperature, the reaction was separated by magnet and the magnetic beads were washed twice with 80% ethanol. The libraries were resuspended in 55 µL Buffer HT and the supernatant was collected. The libraries were quantified using the Qubit Fluorometer and the Qubit dsDNA HS Assay kit (cat. number Q32854, Thermo Fisher Scientific) according to the manufacturer's instructions. Sequencing was performed on the NovaSeq 6000 Sequencing System (Illumina) where sequence data with a target depth of 1,000,000 sequence pairs (2×150 bp) were generated.

Conversion and demultiplexing of the sequence reads were performed with bcl2fastq v2.20. Sequence reads were analysed using the Swedish national pipeline for SARS-CoV-2 analyses, gms-artic (https://github.com/genomic-medicine-sweden/gms-artic/). This analysis is based on the internationally recommended code (https://github.com/connor-lab/ncov2019-artic-nf) developed by the ARTIC Network and Public Health Wales for SARS-CoV-2 sequence data. Sequences were trimmed using Trim Galore ([3]; https://github.com/FelixKrueger/TrimGalore) and then mapped to the SARS-CoV-2 reference genome Wuhan-Hu-1 (accession number MN908947.3) using bwa [4]. Using iVar [5], primer sequences were trimmed, and a consensus sequence was generated for each sample. The criterion for an acceptable sequence result was coverage of at least 95% of the genome at a depth of at least ×10.

1.2.4. Ion Torrent

Twenty samples were sequenced using the Ion Torrent sequencing platform at the Department of Clinical Microbiology, Sahlgrenska University Hospital, Sweden. Extractions were performed with the same method as described above for Illumina sequencing. RNA was prepared according to the protocol for the Ion AmpliSeq SARS-CoV-2 Research Panel (Thermo Fisher Scientific). Reverse transcription was performed with the SuperScript VILO cDNA synthesis kit (cat. number 11754050) on an IonCode 96-well PCR Plate (Thermo Fisher Scientific). The subsequent library preparation and barcoding PCR was performed on the Ion Chef platform with the Ion AmpliSeq Kit for Chef DL8 (cat. number A29024, Thermo Fisher Scientific) according to the manufacturer's instructions. All sample libraries were pooled prior to sequencing, and a final concentration of 30 pM was achieved. Ligation of the barcoded and pooled libraries onto spheres was made with the Ion 510, 520, 530 Kit-Chef (Thermo Fisher Scientific) on the Ion Chef instrument. Libraries were after standard clonal expansion of fragments loaded onto an Ion 530 Chip and the 200 bp read length mode was used for sequencing on the S5 System (XL, Prime; Thermo Fisher Scientific).

Sequencing reads from the Ion Torrent sequenced samples were processed using the Torrent Suite Software for SARS-CoV-2 (Thermo Fisher Scientific) with standard configurations. Briefly, reads were demultiplexed, primer regions trimmed off and mapped to a SARS-CoV-2 reference genome (MN908947.3). Consensus sequences were extracted from the read mappings using the IRMAreport tool (Thermo Fisher Scientific).

1.2.5. MGI

For samples in Örebro, the sequencing was performed at the National Pandemic Centre (NPC), Stockholm, Sweden, and bioinformatically processed for generation of consensus sequences at the Public Health Agency of Sweden. The ATOPlex SARS-CoV-2 full-length genome panel (MGI) was used to construct libraries of short amplicons (159–199 bp) according to the manufacturer's instructions. The RNA sample was converted to cDNA using reverse transcriptase (RT) with random hexamers (MGI). The 20-µL RT reaction mixture contained 10 µL of RNA template, 4 µL of N6 buffer, 5 µL of RT buffer, 12.5 µM of random hexamers, and 1 µL of RT enzyme mix. The RT reaction was performed in a C1000 thermal cycler (Bio-Rad, Hercules, CA, USA) using the program: 10 minutes at 25°C, 30 minutes at 42°C, 15 minutes at 70°C. Lambda phage DNA (200 GC) was added into each sample as a spike-in control to ensure each sample generated sufficient amplification products for sequencing and relative quantification of SARS-CoV-2 RNA (>4 ng/µL). Lambda phage DNA and SARS-CoV-2 primers were co-amplified in the same reaction as follows: DNA/cDNA samples were subjected to two rounds of PCR for target enrichment (first round) and addition of dual barcode (second round). In the first round, the PCR amplification mixture contained 25 µL of PCR Enzyme Mix, 0.5 µL of PCR Clean Enzyme, 4 µL of PCR Primer Pool and 20 µL cDNA. The first- round PCR cycling parameters were 5 minutes at 37°C, 10 minutes at 95°C, 13 cycles at 95°C for 10 seconds, 64°C for 1 minute, 60°C for 1 minute, and 72°C for 10 seconds, followed by a final extension step at 72°C for 2 minutes performed on a C1000 thermal cycler. The first-round PCR products were then purified using 1.2 ×60 µL clean magnetic beads. In the second round the PCR amplification mixture contained 25 µL of PCR Enzyme Mix, 0.5 µL of PCR Clean Enzyme, 1 µL of PCR additive, 2 µL of PCR block (259 sets of barcoded SARS-CoV-2 primers, each targeting a different region of about 200 bp to encompass the entire

genome (accession MN908947.3), 10 sets of Lambda Phage DNA primers; and four sets of primers targeting the human Glyceraldehyde-3-phosphate dehydrogenase (GAPDH) gene for human DNA/RNA contamination control into the purified PCR products from the first round. The second-round PCR was performed under the same cycling parameters as that of the first-round PCR, except that 27 PCR cycles were used. The second-round PCR products were also purified using 0.9 ×45 μL clean magnetic beads. After bead-based purification, the second-round PCR products were quantified with the Qubit dsDNA High Sensitivity Assay kit (Thermo Fisher Scientific) to confirm the required concentration of ≥4 ng/μL.

Short amplicon libraries from each sample were pooled at equimolar levels and subjected to single-stranded circular DNA library preparation with the MGIEasy Dual Barcode Circularization kit (MGI) to obtain circularized DNA molecules. These molecules were subsequently digested to form circularized single-strand DNA and then subjected to rolling circle amplification to generate DNA nanoballs-based libraries, which were then subsequently sequenced on the MGI-2000 platform (MGI) according to the manufacturer's instructions.

The sequences from MGI-2000 were assembled at the Public Health Agency of Sweden using a validated pipeline.

1.3. Phylogenetics trees

The Augur workflow was based on the SARS-CoV-2 workflow, git-version d73ca130ee5320d86726fab1524d2d443fdfd907.

In the down-sampling for the time-scaled tree, the subsampling schemes for the context sequences, the dataset of 129,913 non-Swedish sequence was sampled randomly to obtain 500 European and 500 non-European sequences with priority for proximity to Swedish sequences, in addition to 100 European and 100 non-European random sequences.

For the generation of a variant call format (.vcf) file of the sequences, sites were masked according to recommendations in the documentation using the file downloaded from https://raw.githubusercontent.com/W-L/ProblematicSites_SARS-CoV2/master/problematic_sites_sarsCov2.vcf (file date: 2021-10-27).

2. Figures and tables

The SARS-CoV-2 case numbers in the Stockholm Region in Figure 2 were obtained from the records at the regional Infectious Disease Control Authority. These case numbers are not identical to the case numbers of the Public Health Agency of Sweden due to slightly different methods for data cleaning.

Figures 2 and 4 were generated using the matplotlib package [6]. Figure 3 was created by modifying a map of Sweden from Wikimedia Commons using Inkscape v 1.2.2 (Inkscape Project). The phylogenetic trees were generated using the export tool in Auspice. Figures 6 and S1 were generated in Stata 15 IC (StataCorp LLC).

2.1. Table S1

Timeline of cases and major events in Sweden during the early phase of the SARS-CoV-2 pandemic

Week in 2020	Diagnosed Swedish cases	Deaths due to COVID-19 in Sweden	Date	Event
3	0	0	16 January	The PHAS establishes SARS-CoV-2 diagnostics. Test indication: persons with respiratory symptoms, fever, and cough and travel history to Wuhan, China.
4	0	0	20 - 26 January	
5	1	0	31 January	First Swedish case diagnosed, travel history to Wuhan.
			1 February	The Swedish government classifies SARS-CoV-2 as a disease dangerous to society.
6	0	0	5 February	Persons returning from affected areas in China urged to be attentive to symptoms. Symptomatic persons instructed to contact health care.
7-8	0	0	10 - 23 February	
9	13	0	24 February	Update of risk areas by PHAS: China, South Korea, northern Italy, and Iran.
			25 February	The PHAS assesses the risk of generalized transmission in Sweden to be low.
			26 February	A second Swedish case, travel history to northern Italy.
			27 February	Five cases in three Swedish regions diagnosed, all travel-related, either directly or indirectly by close contact with a known case.
10	211	0	2 March	Risk of new cases infected with COVID-19 in Sweden is assessed as very high and risk of a generalized transmission in Sweden assessed as moderate.
			3 March	Testing recommended for persons developing symptoms within 14 days after having visited northern Italy.
			4 March	All known cases linked to travel abroad. The PHAS recommends clinical laboratories to establish SARS-CoV-2 testing for patients with respiratory symptoms of unknown cause.
11	838	5	9 March	The PHAS extends the test recommendation to symptomatic people returning from Tyrol region, Austria. Travelers who develop symptoms within 14 days of return are recommended to stay at home. The risk of spread in Sweden is assessed as moderate, with no signs of ongoing community transmission.
			10 March	The PHAS assesses there are signs of community transmission in the regions of Stockholm and Västra Götaland Regions and raises the risk level to the highest for the rest of Sweden. In case of respiratory symptoms limited contact with others is recommended, especially for those working in elderly care. It is also recommended that relatives avoid unnecessary visits to hospitals and nursing homes.
			11 March	The first fatal case in Sweden.
			13 March	The strategy to target testing to returning travellers from risk regions is stopped. Anyone with respiratory symptoms is urged to stay home. General testing for SARS-CoV-2 of symptomatic persons is assessed as not indicated. The government limits public gatherings to 500 people.

Table S1 cont.

**Week in 2020**	**Diagnosed Swedish cases**	**Deaths due to COVID-19 in Sweden**	**Date**	**Event**
12	924	44	16 March	The PHAS recommends employers to facilitate work from home for their employees if possible.
			17 March	The PHAS recommends remote learning for upper secondary schools, universities, polytechnics, and municipal adult education.
13	1,957	190	23 March	PHAS urges people aged 70 and older to stay at home.
			24 March	The PHAS draws up regulations and general advice to prevent the spread of infection at catering establishments. Among other things, restaurants, bars and cafes must ensure that crowding does not occur.
14	3,229	451	1 April	The government ban on visitors to nursing homes comes into effect.

Events in the timeline and numbers of detected cases and deaths due to COVID-19 according to public data from the Public Health Agency of Sweden [7]. PHAS: Public Health Agency of Sweden.

2.2. Table S2

Information about sequences from the GISAID repository.

SUPPLEMENTAL TABLE

Data Availability

GISAID Identifier: EPI_SET_231220zv
doi: 10.55876/gis8.231220zv

All genome sequences and associated metadata in this dataset are published in GISAID's EpiCoV database. To view the contributors of each individual sequence with details such as accession number, Virus name, Collection date, Originating Lab and Submitting Lab and the list of Authors, visit 10.55876/gis8.231220zv

Data Snapshot

- EPI_SET_231220zv is composed of 147,007 individual genome sequences.
- The collection dates range from 2019-12-05 to 2022-04-14;
- Data were collected in 157 countries and territories;
- All sequences in this dataset are compared relative to hCoV-19/Wuhan/WIV04/2019 (WIV04), the official reference sequence employed by GISAID (EPI_ISL_402124). Learn more at https://gisaid.org/WIV04.

2.3. Table S3.

Cases and sequences in Sweden

Region	Confirmed Cases	Sequences	Sequences per 100 cases	Fraction of sequences (%)
Blekinge	218	0	0	0
Dalarna	1,227	40	3.3	2.4
Gävleborg	1,304	7	0.5	0.4
Gotland	82	0	0	0
Halland	802	69	8.6	4.1
Jämtland	734	11	1.5	0.7
Jönköping	1,566	10	0.6	0.6
Kalmar	362	14	3.9	0.8
Kronoberg	857	0	0	0
Norrbotten	437	17	3.9	1.0
Örebro	1,845	218	11.8	13.0
Östergötland	2,064	45	2.2	2.7
Skåne	1,770	28	1.6	1.7
Sörmland	1,675	20	1.2	1.2
Stockholm	13,204	823	6.2	48.9
Uppsala	1,965	56	2.8	3.3
Värmland	540	30	5.6	1.8
Västerbotten	500	18	3.6	1.1
Västernorrland	756	25	3.3	1.5
Västmanland	1,317	17	1.3	1.0
Västra Götaland	6,084	232	3.8	13.8
No data	NA	4	NA	0.2
**Total**	**39,309**	**1,684**	**4.3**	**100**

Number of sequences per Swedish region in relation to the number of confirmed cases until 1 June 2020, according to public data from the Public Health Agency of Sweden [8]. Information about region was missing for four sequences. NA: not applicable.

1.1. Figure S1.

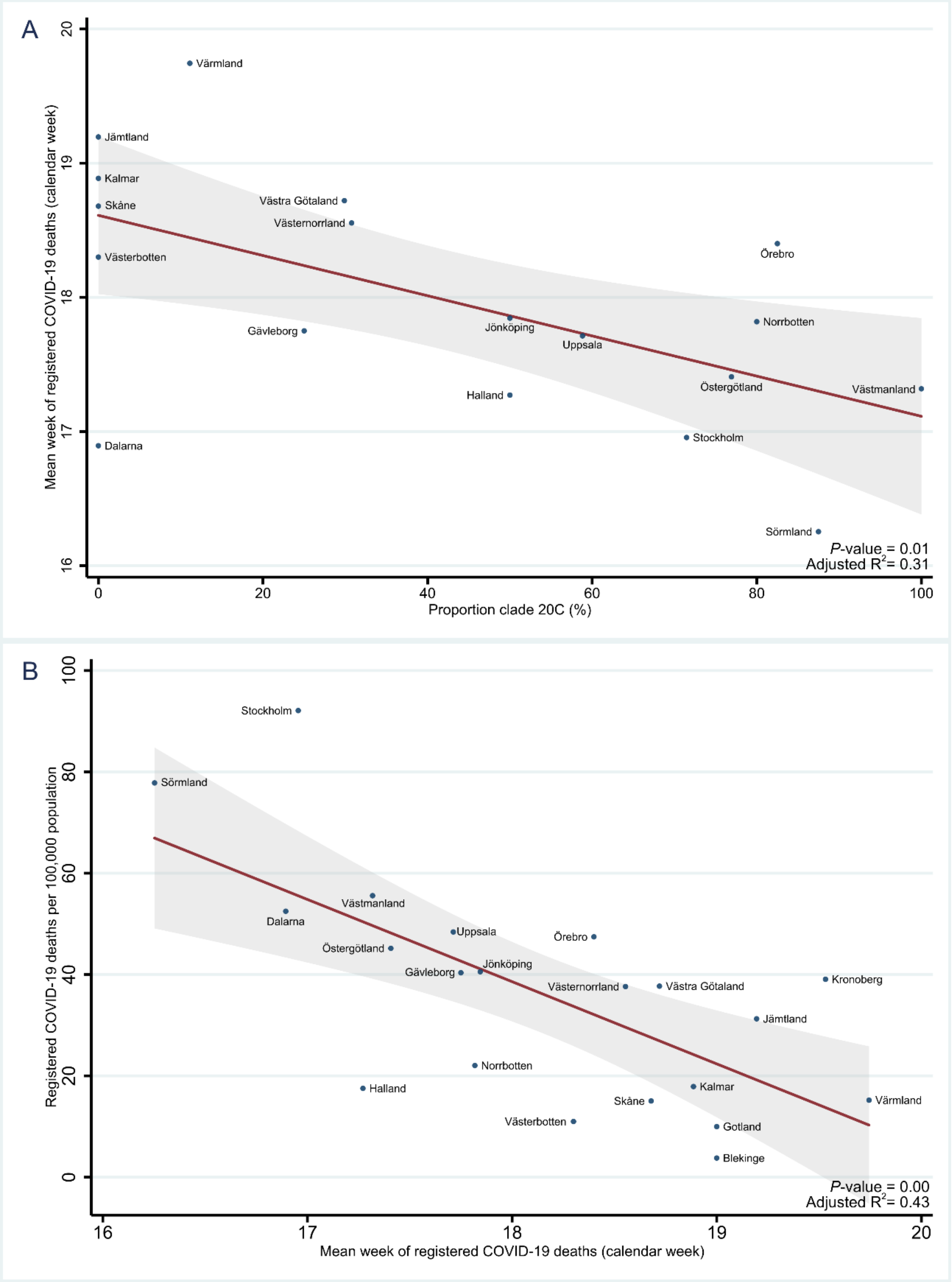

(A) The mean date of registered deaths due to COVID-19 plotted against the proportion of clade 20C among cases until 1 June 2020 exposed in Sweden. A higher proportion of clade 20C correlated with an earlier mean date of deaths. All cases were analysed with exposure status as reported.
(B) The cumulative number of registered deaths due to COVID-19 in Sweden until 7 June 2020 plotted against the mean date of registered deaths due to COVID-19. An earlier mean date of registered deaths correlated with a higher cumulative number of registered deaths.
The mean week of reported COVID-19 deaths in a region was calculated as a weighted mean, where the date (calendar week) was weighted by the number of reported deaths. Line = fitted mean of linear regression; shaded area = 95% confidence interval of the predicted mean.

References

1. Ringlander J, Fingal J, Kann H, Prakash K, Rydell G, Andersson M, et al. Impact of ADAR-induced editing of minor viral RNA populations on replication and transmission of SARS-CoV-2. Proceedings of the National Academy of Sciences. 2022;119(6):e2112663119.
2. Corman VM, Landt O, Kaiser M, Molenkamp R, Meijer A, Chu DK, et al. Detection of 2019 novel coronavirus (2019-nCoV) by real-time RT-PCR. Euro surveillance : bulletin Europeen sur les maladies transmissibles = European communicable disease bulletin. 2020;25(3).
3. Martin M. Cutadapt removes adapter sequences from high-throughput sequencing reads. EMBnet journal. 2011;17(1):10-2.
4. Li H, Durbin R. Fast and accurate short read alignment with Burrows-Wheeler transform. Bioinformatics. 2009;25(14):1754-60.
5. Grubaugh ND, Gangavarapu K, Quick J, Matteson NL, De Jesus JG, Main BJ, et al. An amplicon-based sequencing framework for accurately measuring intrahost virus diversity using PrimalSeq and iVar. Genome Biol. 2019;20(1):1-19.
6. Hunter JD. Matplotlib: A 2D graphics environment. Computing in science & engineering. 2007;9(03):90-5.
7. The Public Health Agency of Sweden. När hände vad under pandemin? [When did what happen during the pandemic?] [Available from: https://www.folkhalsomyndigheten.se/smittskydd-beredskap/utbrott/aktuella-utbrott/covid-19/nar-hande-vad-under-pandemin/.
8. The Public Health Agency of Sweden. Bekräftade fall av covid-19 i Sverige [Confirmed cases of covid-19 in Sweden] [Available from: https://www.folkhalsomyndigheten.se/smittskydd-beredskap/utbrott/aktuella-utbrott/covid-19/statistik-och-analyser/bekraftade-fall-i-sverige/.
